# Supplementary material for: Association of Dietary Vitamin C Consumption with Serum Klotho Concentrations
Source: Foods. 2023 Nov 23;12(23):4230. doi: 10.3390/foods12234230 (PMC10706666; doi:10.3390/foods12234230)
Supplement: Supplementary file 1 [file foods-12-04230-s001.zip › Supplementary Figure S1.pdf]

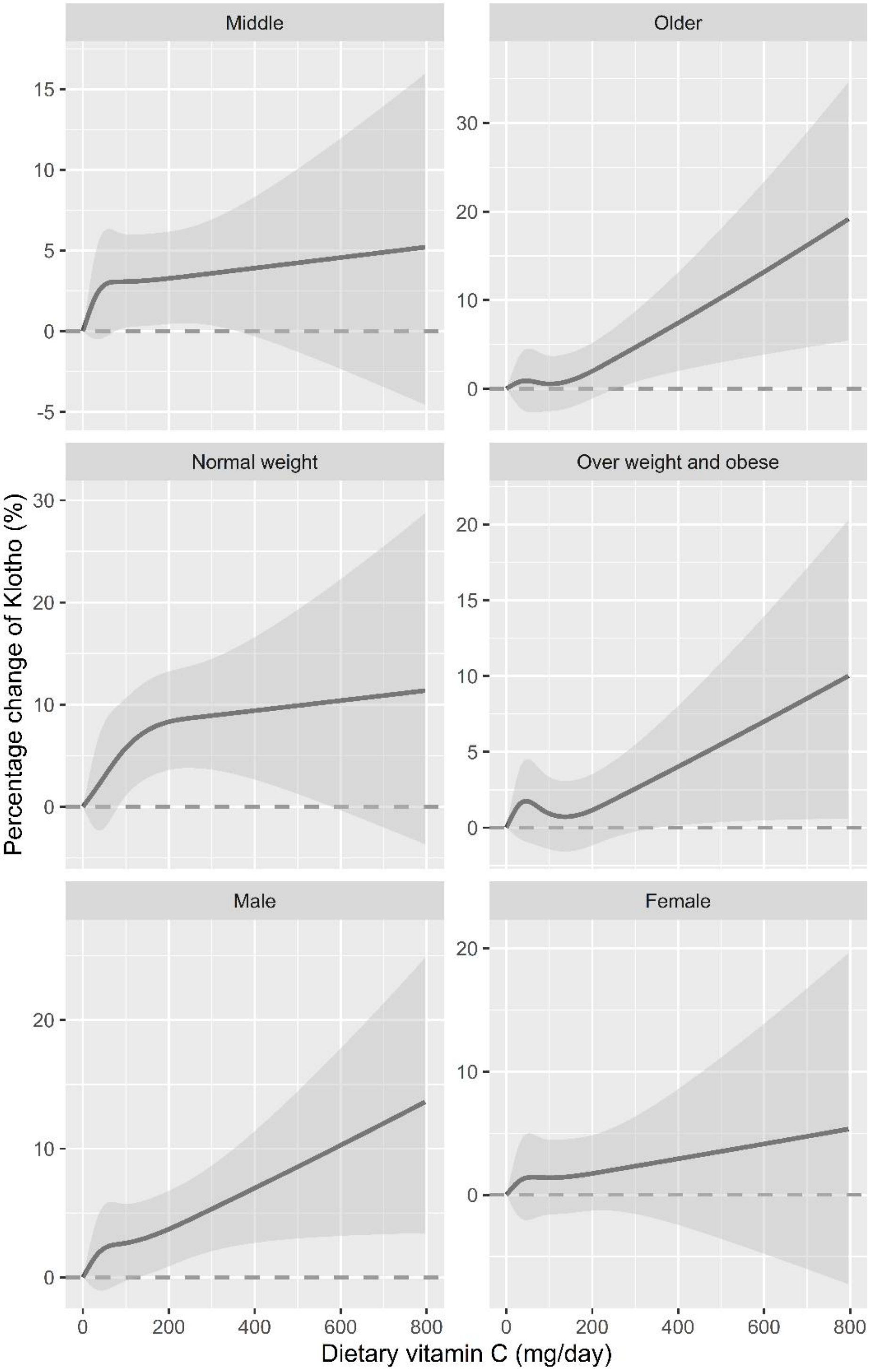

Supplementary Figure S1. The dose-response relationship between dietary vitamin C intakes and percent change of Klotho concentration in the subgroup analysis (Age: <60 (Middle), ⩾60 (Older); BMI: <25 (Normal weight), ⩾25 (Overweight and obese); Sex: Male, Female). Point value estimation (solid line) and 95% confidence interval calculation (dashed line) were estimated by a restrictive cubic spline analysis model, knotted at the 5th, 35th, 65th, and 95th percentiles. The model was adjusted for age, sex, BMI, PIR, education attainment, ethnicity, serum cotinine, alcohol drinking, diabetes or not, hypertension or not, eGFR, and dietary energy intake.
